# A New Risk Prediction Model for the Assessment of Myocardial Injury in Elderly Patients Undergoing Non-Elective Surgery

**DOI:** 10.3390/jcdd12010006

**Published:** 2024-12-26

**Authors:** Vedat Cicek, Mert Babaoglu, Faysal Saylik, Samet Yavuz, Ahmet Furkan Mazlum, Mahmut Salih Genc, Hatice Altinisik, Mustafa Oguz, Berke Cenktug Korucu, Mert Ilker Hayiroglu, Tufan Cinar, Ulas Bagci

**Affiliations:** 1 Machine & Hybrid Intelligence Lab, Department of Radiology, Northwestern University, Chicago, IL 60611, USA; ulasbagci@gmail.com; 2 Sultan II. Abdulhamid Han Training and Research Hospital, Department of Cardiology, Health Sciences University, 34668 Istanbul, Turkey; babaoglumert11@gmail.com (M.B.); dr.sametyavuz@gmail.com (S.Y.); hatice.altinisik98@gmail.com (H.A.); drmustafaoguz@hotmail.com (M.O.); 3 Van Training and Research Hospital, Department of Cardiology, Health Sciences University, 65300 Van, Turkey; faysalsaylik@gmail.com; 4 Sultan II. Abdülhamid Han Training and Research Hospital, Department of General Surgery, Health Sciences University, 34668 Istanbul, Turkey; a.furkan.mazlum@gmail.com (A.F.M.); mslhgenc@gmail.com (M.S.G.); 5 Department of Internal Medicine, Rutgers\Robert Wood Johnson Barnabas Health, Jersey City Medical Center, Jersey City, NJ 07302, USA; cenkorucu@gmail.com; 6 Department of Cardiology, Dr. Siyami Ersek Cardiovascular and Thoracic Surgery Research and Training Hospital, 34668 Istanbul, Turkey; mertilkerh@gmail.com; 7 School of Medicine, University of Maryland, Baltimore, MD 21201, USA; drtufancinar@gmail.com

**Keywords:** cardiac revised index, elderly, myocardial injury, non elective surgery, pre-op risk

## Abstract

**Background:** Currently, recommended pre-operative risk assessment models including the revised cardiac risk index (RCRI) are not very effective in predicting postoperative myocardial damage after non-elective surgery, especially for elderly patients. **Aims:** This study aimed to create a new risk prediction model to assess myocardial injury after non-cardiac surgery (MINS) in elderly patients and compare it with the RCRI, a well-known pre-operative risk prediction model. **Materials and Methods:** This retrospective study included 370 elderly patients who were over 65 years of age and had non-elective surgery in a tertiary hospital. Each patient underwent detailed physical evaluations before the surgery. The study cohort was divided into two groups: patients who had MINS and those who did not. **Results:** In total, 13% (48 out of 370 patients) of the patients developed MINS. Multivariable analysis revealed that creatinine, lymphocyte, aortic regurgitation (moderate-severe), stroke, hemoglobin, ejection fraction, and D-dimer were independent determinants of MINS. By using these parameters, a model called “CLASHED” was developed to predict postoperative MINS. The ROC analysis comparison demonstrated that the new risk prediction model was significantly superior to the RCRI in predicting MINS in elderly patients undergoing non-elective surgery (AUC: 0.788 vs. AUC: 0.611, *p* < 0.05). **Conclusions:** Our study shows that the new risk preoperative model successfully predicts MINS in elderly patients undergoing non-elective surgery. In addition, this new model is found to be superior to the RCRI in predicting MINS.

## 1. Introduction

Rising life expectancy has led to a global surge in the elderly population, with significant implications for healthcare systems. In the United States, for example, over half of all emergencies surgical procedures are performed on older adults [1,2]. However, these patients are at an increased risk of post-surgical complications and mortality due to factors like frailty, multimorbidity, diminished physiological reserve, and polypharmacy.

Myocardial injury is demonstrated to be one of the primary reasons for increased peri-operative and post-operative mortality in elderly patients. A new concept termed myocardial injury after non-cardiac surgery (MINS) has been recently introduced to encompass not only infarctions but also other perioperative myocardial injuries of prognostic significance attributed to ischemia [3,4]. MINS excludes perioperative myocardial injury stemming from a documented non-ischemic etiology such as pulmonary embolism, sepsis, and acute decompensated heart failure. It has been shown that the presence of MINS is correlated with elevated mortality rates within the first 30 days post-surgery and prolonged inpatient stays [5].

Current guidelines advocate for preoperative risk stratification using scoring systems like the Revised Cardiac Risk Index (RCRI) to predict perioperative and postoperative cardiac complications in elderly patients undergoing non-cardiac surgery [6]. However, the efficacy of these models, particularly in emergency settings, remains debatable [7]. This study aimed to develop and validate a new risk prediction MINS model specifically for major non-elective surgery in elderly patients, comparing its efficacy with the established RCRI.

## 2. Materials and Methods

### 2.1. Patients

This retrospective study was planned by examining the archive records of a tertiary hospital. In total, 370 elderly patients who were 65 and older comprised the study cohort. In this study, three different groups of elderly patients who had non-elective surgery due to hip fracture, acute abdominal pathologies, and neurosurgical pathologies were included. Patients who were under 65 years of age or who had type 1 myocardial infarction, acute decompensated heart failure, acute pulmonary embolism, acute renal failure, sepsis, stroke, and atrial fibrillation with rapid ventricular response were eliminated from this study. Patients who underwent emergency surgery (within 1 h) without a pre-operative cardiology consultation request were excluded from this study. The hip fracture patient group underwent procedures under spinal anesthesia unless contraindicated, while other patient groups were operated on under general anesthesia. Demographic characteristics and risk factors of all patients were recorded. Detailed examinations were conducted, including physical examination, electrocardiography, and transthoracic echocardiography, before the surgery. All lab results and treatment protocols were also documented. The Ethics Committee authorized this study, which was carried out in conformity with the principles of the Helsinki Declaration (decision number: 23\607).

### 2.2. MINS Definition

Perioperative myocardial injury can be defined as a process leading to cardiomyocyte damage, evidenced by an elevated cardiac injury biomarker. The Vascular Events in Noncardiac Surgery Patients Cohort Evaluation (VISION) study defined a novel condition termed MINS. MINS refers to myocardial cellular damage occurring within 30 days of non-cardiac surgery attributed to ischemic causes [8]. Non-ischemic contributors, such as sepsis or pulmonary embolism, are excluded from this definition. Fundamentally, MINS represents an ischemic perioperative myocardial injury with significant prognostic implications, encompassing both perioperative myocardial injury and infarction. Large-scale cohort studies indicate that perioperative myocardial infarction (PMI) comprises only 20–40% of MINS cases, depending on the cardiac troponin assay used [9].

The 2018 Task Force for the Universal Definition of Myocardial Infarction defines myocardial injury as an elevation of cardiac troponin values, with at least one measurement exceeding the 99th percentile upper reference limit. Acute myocardial infarction (MI) represents a subset of acute myocardial injury characterized by evidence of myocardial ischemia resulting in cell death. MI is diagnosed based on a rise and fall in troponin levels accompanied by at least one of the following criteria: (1) ischemic symptoms such as chest pain or pressure, (2) new ischemic changes on electrocardiography (e.g., ST-segment elevation or depression, T-wave inversion), (3) development of pathological Q waves, (4) new regional wall motion abnormalities or evidence of myocardial loss on imaging modalities such as echocardiography or myocardial perfusion imaging, and (5) identification of a coronary thrombus via angiography or autopsy [10,11].

Based on these criteria, MINS was defined by at least 1 postoperative cardiac troponin concentration that exceeded the 99th percentile upper reference limit of the troponin assay (the Roche Cobas high sensitive Troponin T assay is used in our hospital) as a result of a presumed ischemic mechanism without overt non-ischemic causes. In patients whose initial troponin value exceeded the 99th percentile, at least a >20% increase in subsequent troponin concentration was considered MINS.

### 2.3. Non-Elective Surgery Definition

The NEST (Non-Elective Surgery Triage) classification system was used to categorize this cohort of patients. NEST is a structured framework designed to prioritize surgical interventions based on clinical urgency, aiming to optimize resource allocation and patient outcomes in non-elective settings. It categorizes procedures into three primary tiers: emergency surgeries (NEST 1 and NEST 2 should undergo surgery within 1 h), which are immediate and life-saving interventions that must be performed without delay; urgent surgeries (NEST 3 and NEST 4 patients are recommended to be operated on within 12), which are required within 12 h to address conditions that could deteriorate rapidly if untreated; and semi-urgent surgeries NEST 5 patients should receive surgical intervention within 48 h), which, while medically necessary, can be scheduled within a few days without posing an immediate threat to the patient’s survival [12]. This system has been instrumental in high-demand environments, such as during mass casualty events or resource-limited situations, ensuring timely care for conditions ranging from traumatic injuries, such as spinal fixation and hip fractures, to life-threatening abdominal emergencies, like bowel obstructions or a perforated viscus [13]. The implementation of NEST classification facilitates decision-making and improves surgical care efficiency by balancing clinical urgency with resource constraints, ultimately enhancing outcomes for non-elective surgical patients [14]. The patient group in this study was classified under the NEST system as requiring urgent surgeries to be performed within 24 to 48 h.

## 3. Statistical Analysis

The statistical analyses were computed using R statistical software, version 4.1.2. The Kolmogorov–Smirnov test was performed to see if the samples were normally distributed. The categorical data were shown using percentages and numbers. Depending on the situation, either Fisher’s exact test or the 2 test was used to compare categorical variables between groups. The continuous data were expressed as the mean (SD) for normal distributions and median (interquartile range (IQR)) for non-normal distributions. The independent Student’s *t*-test and Mann–Whitney U tests were used to compare continuous variables between the groups. Lasso penalized regression was used to select variables for the multivariable model using the minimum lambda value for penalization to avoid overfitting. Thereafter, lymphocyte, hemoglobin, serum creatinine, D-dimer, left ventricular (LV) ejection fraction, cerebrovascular accident, and moderate-to-severe aortic regurgitation (AR) were used to create a multivariable predictive model. Multicollinearity was assessed using tolerance (0.1) and VIF (variance inflation factor > 3) values. Logistic regression with penalized shrinkage was employed to detect independent associations of variables with post-op myocardial injury. Based on X2 values, the variables in the multivariable model were ordered according to their importance. A nomogram based on the multivariable model was created to predict the risk of post-op myocardial injury. Internal validation with the bootstrapping method using 300 iterations was used for model validation and a calibration plot was drawn for the generalizability of the nomogram. Finally, to compare the discriminative capacities of the developed model and the RCRI for patients with post-operative myocardial injury, the receiver–operating characteristics (ROC) curve comparison was presented. The data analysis was performed using a 2-sided *p* < 0.05 and a 95% confidence interval (CI).

## 4. Results

In all, 13% (48 out of 370 patients) of the patients developed MINS. We divided the study cohort into two groups; patients who had MINS and those who did not. Table 1 shows the demographic characteristics and laboratory data of all patients. Accordingly, 245 of the patients had hip surgery, 64 patients had neurosurgical surgery, and 61 patients had major surgery due to acute abdominal pathologies. There was no statistically significant difference between the two groups in terms of age, surgery type, or gender. Urea was higher and hemoglobin was lower in elderly patients who developed MINS. In terms of echocardiography data, the LV ejection fraction was lower, the left atrium was larger, and aortic regurgitation (AR) was observed more frequently in the group that developed MINS. In-hospital stays and in-hospital mortality rates were higher in elderly patients who developed MINS. In-hospital mortality occurred in 52.1% (25 patients) of MINS+ patients, and it was observed in 32.1% (9 patients) of patients who developed MINS after hip fracture surgery, 81.8% (9 patients) of patients who developed MINS after neurosurgical procedures, and 77.8% (7 patients) of patients who developed MINS after abdominal surgery. RCRI was higher in the group that developed MINS. Among the 370 patients included in our cohort, 62 (17%) had troponin levels above the 99th percentile (troponin > 14 ng/L) at the time of admission. Of these 62 patients, 34 (71% of MINS+) later developed postoperative MINS+, while 28 (9% of MINS-) were MINS-patients. Elevated troponin levels at admission were found to be statistically significantly associated with the development of postoperative MINS (*p* < 0.001). The admission troponin levels in patients with chronic kidney disease (CKD) were 19 (3–37), whereas in those without CKD, the admission troponin levels were 4 (2–12). Troponin levels at admission were significantly higher in patients with CKD compared to those without (*p* = 0.001).

**Figure 1:**
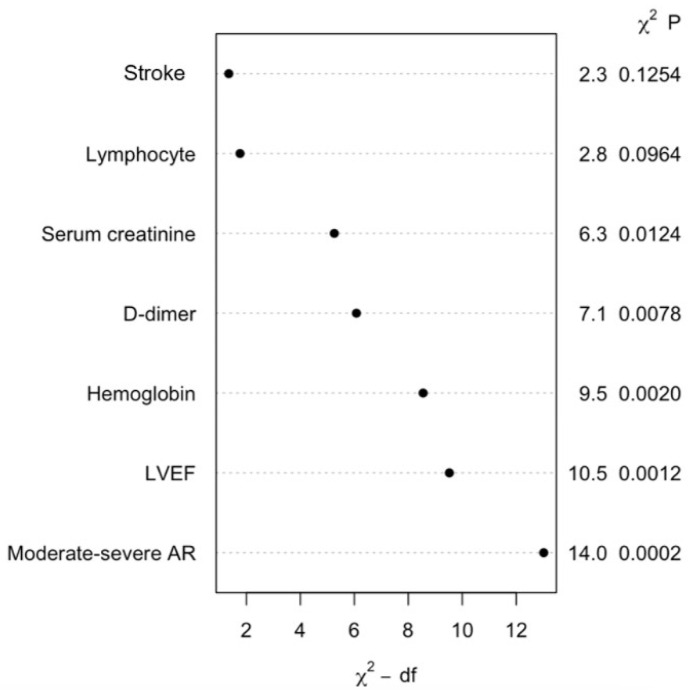
Efficient parameters of the CLASHED model developed for MINS prediction after non-elective surgery in elderly patients.

**Figure 2:**
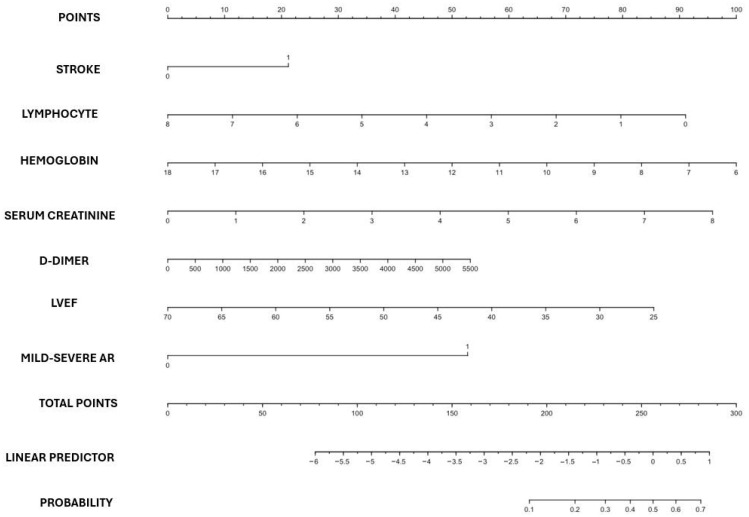
The nomogram of the CLASHED model developed for MINS prediction after non-elective surgery in elderly patients.

Table 2 shows the independent predictors of MINS as a result of multivariable logistic regression. Creatinine, lymphocyte, moderate–severe AR, stroke history, hemoglobin, LV ejection fraction, and D-dimer were independent predictors of MINS (Figure 1). A model termed CLASHED was created to predict MINS in elderly patients undergoing non-elective surgery developed with the data shown in Figure 2.

**Figure 3:**
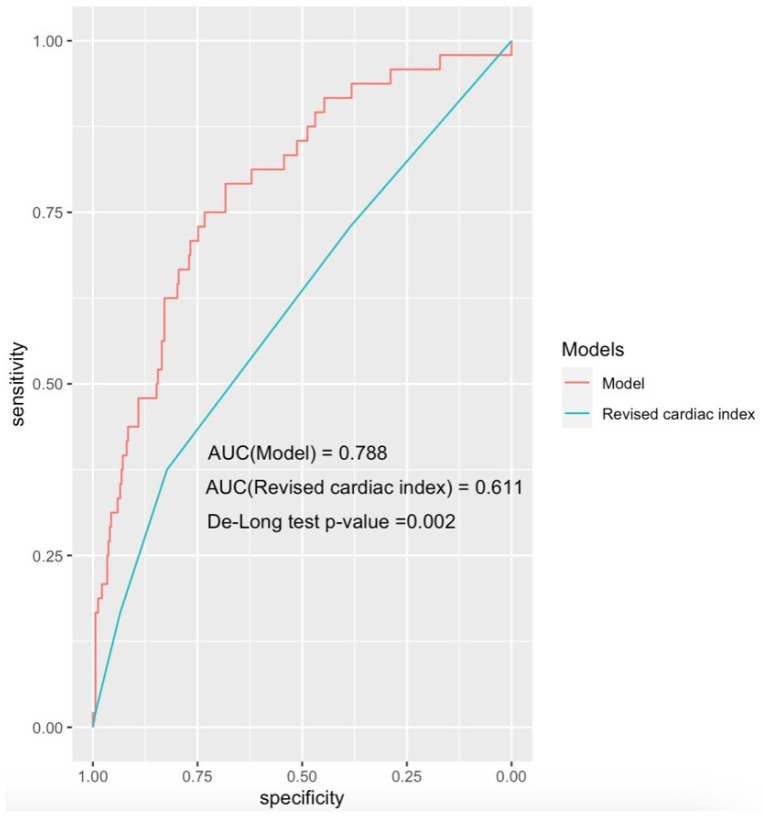
Performance of the CLASHED model and comparison with the Revised Cardiac Risk Index in non-elective surgery for elderly patients.

**Figure 4:**
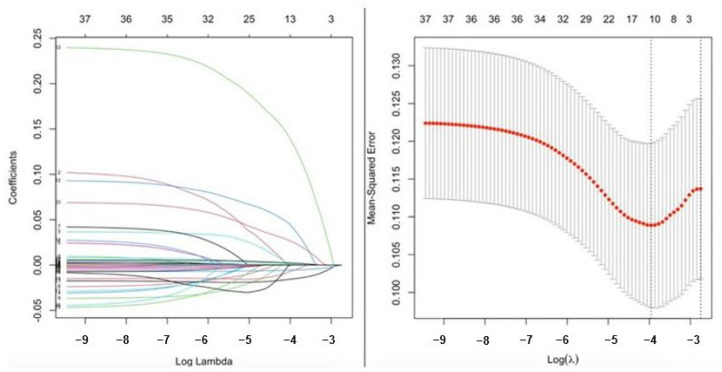
The sorts of contributions of variables in the multivariable predictive model based on chi-square values. Each colorful line represents the regression coefficients of different variables, which are penalized to zero during Lasso regularization to select variables with non-zero for the final the multivariable model.

**Figure 5:**
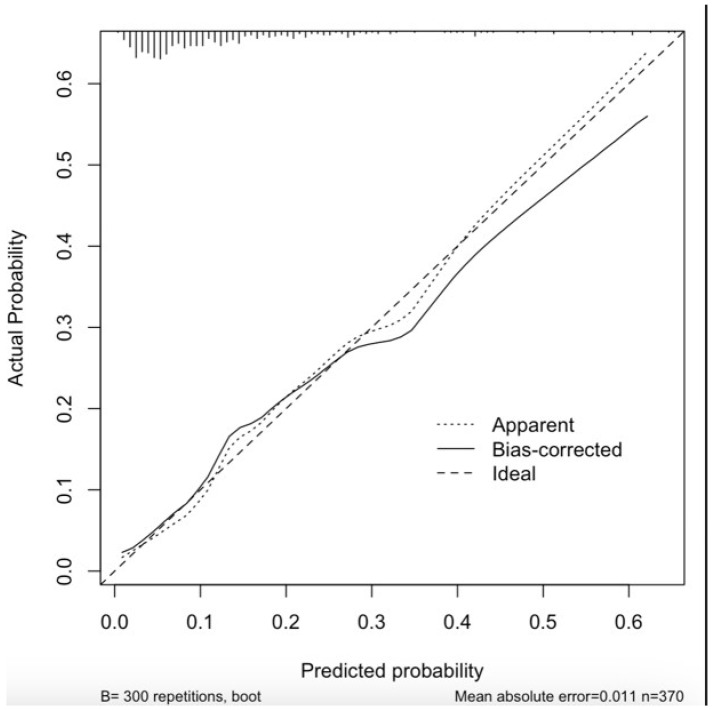
Calibration plot of the CLASHED model.

The ROC analysis showed that the ‘CLASHED’ model had an area under the curve (AUC) of 0.788 and the RCRI had an AUC of 0.611. The CLASHED model was significantly superior to the RCRI in predicting MINS (*p* < 0.05) (Figure 3). The sorts of contributions of variables in the multivariable predictive model were based on chi-square values (Figure 4). Internal validation showed that the generalizability of the prediction model was good, as shown in the calibration plot (Figure 5). The ROC analysis showed that the ‘CLASHED’ model had an area under the curve (AUC) of 0.788, while the RCRI had an AUC of 0.611. The CLASHED model was significantly superior to the RCRI in predicting MINS (*p* < 0.05) (Figure 3). The contributions of variables in the multivariable predictive model were based on chi-square values (Figure 4). Internal validation demonstrated that the generalizability of the prediction model was good, as shown in the calibration plot (Figure 5).

In our model with seven different parameters, the result for each parameter evaluates for the patient is marked. The corresponding projections of the marked points in the first row (“Points”) is summed one by one. The total is then marks in the lower row as “Total Points”, and the corresponding vertical projection of the marked point in the “Probability” row indicates the MINS development risk.

For example, a 72-year-old patient scheduled for acute abdomen surgery with the following parameters: a history of stroke (20 points), hemoglobin level of 10 g/dL (65 points), lymphocyte level of 2 × 10³/µL (67 points), creatinine level of 1.5 mg/dL (17 points), D-dimer level of 2000 ng/mL (18 points), LV ejection fraction of 35% (68 points), and mild AR (0 points), had a total of 255 points. The “Probability” of the total points was 0.5, indicating a 50% risk of developing post-op MINS.

## 5. Discussion

The world’s population is aging rapidly, with the proportion of individuals over 65 exceeding 17% in the United States alone [15]. This demographic shift translates to a growing number of emergency surgical interventions in this vulnerable population, often leading to poorer outcomes [16]. Studies have shown that elderly patients (≥65) undergoing non-elective surgery experience significantly higher mortality rates (12.5%) compared to those undergoing elective procedures (2.6%) [17]. Myocardial ischemia is recognized as a major contributor to postoperative mortality in this group. Our study confirms this association, showing a higher risk of death in patients experiencing major peri-operative non-cardiac ischemia (MINS). Notably, non-elective surgery in this population frequently involves urgent interventions for intra-abdominal pathologies, trauma, and vascular conditions [18]. We demonstrate that our newly developed model effectively predicts MINS risk in these specific surgical contexts.

Previous studies showed that some predictors of MINS include history of atherosclerotic cardiovascular disease, cerebrovascular disease, and related risk factors such as diabetes and hypertension [19,20]. Moreover, some biomarkers such as low hemoglobin, elevated D-dimer, and lymphocyte count have been shown to be correlated with MINS [21]. Furthermore, high creatinine levels and low LV ejection fractions could be considered additional risk factors for MINS. As our model included these parameters, it was not unexpected to observe that our model successfully predicted MINS in elderly patients.

RCRI is a widely used perioperative cardiac risk tool to help determine which patients are at a high risk of perioperative MI or cardiac arrest. The RCRI relies on six variables (history of ischemic heart disease, heart failure, stroke, insulin-dependent diabetes, chronic kidney disease, high-risk surgery) to categorize individuals into low-, intermediate-, or high-risk groups for perioperative cardiac complications during noncardiac surgeries [22]. This score has several limitations. Its limitations, particularly in specific subgroups, have been increasingly recognized. For instance, a single North American study demonstrated that the RCRI performs poorly in patients with kidney failure undergoing surgery [23]. Insulin-dependent diabetes mellitus, while traditionally considered a significant risk factor, was not a significant predictor in a multivariate analysis in this context [24]. This underscores the need to refine the RCRI to incorporate more robust predictors such as cardiac biomarkers. D-dimer, high-sensitivity troponin T, and B-type natriuretic peptide have shown substantial prognostic value beyond the RCRI [25,26,27]. Age is a critical independent predictor of cardiovascular events. In older patients, the RCRI’s ability to predict major postoperative cardiovascular events has been shown to be relatively poor [28,29]. The lack of dynamic compensatory ability of the cardiac vascular system among older individuals may amplify the risk associated with prolonged exposure to clinical risk factors. Studies, including a large Danish national cohort, have demonstrated that advanced age (≥70 years) significantly increases the risk of major adverse cardiovascular events [30]. This finding highlights the need for age-adjusted risk stratification models. Additionally, peripheral artery disease (PAD) appears to be a stronger predictor of perioperative cardiac. PAD has an independent association with cardiac injury in noncardiac surgery [31]. Stroke risk prediction is particularly challenging due to its multifactorial nature, influenced by hemodynamic instability, intracranial vessel morphology, and comorbidities. In patients undergoing noncardiac surgery, Patients with a history of stroke or TIA are at a higher risk of a perioperative stroke and subsequent poor clinical outcomes and cardiac complications [32]. While RCRI can make a moderate distinction in elective surgeries, it does not have an evidence-based recommendation for non-elective surgeries [33]. The CLASHED model that was developed by using D-dimer, stroke history, hemoglobin, lymphocyte, serum creatinine, and echocardiographic parameters in elderly patients has been shown to be statistically significantly superior to RCRI, thus creating a model option for those undergoing non-elective surgery.

In [34], which included high-risk patients receiving non-elective surgery, patients with MINS had a short-term mortality of 12% at 30 days and a one-year mortality of 25%. In patients undergoing non-cardiac surgery, MINS should be considered a changeable outcome, particularly in emergency situations. We considered that by using our new risk prediction model, elderly patients at a high risk of MINS could be managed with goal-directed hemodynamic therapy, and cardio-protective anesthesia could be applied to reduce the development of post-operative MINS among these patients.

MINS is a common yet often asymptomatic complication following non-elective surgery and serves as an independent predictor of increased mortality in operative patients [35]. Over recent years, it has been observed that patients undergoing non-elective surgeries frequently experience clinically silent MINS, evidenced by elevated troponin levels [36]. O’Hara et al. reported that more than 16% of elderly patients with hip fractures presented with elevated admission troponin levels [37]. Similarly, Hietala et al. found that in hip fracture patients, more than half of the troponin elevations occurred prior to surgery. Consistent with the literature, our cohort revealed that 17% of patients had troponin levels above the 99th percentile, and 71% of those with MINS already exhibited elevated troponin levels at the time of admission [38]. The non-elective surgery cohort in our study predominantly consisted of patients classified within the NEST categories 3 to 5. Hip fracture patients were classified as NEST 3–5, while patients undergoing acute abdominal surgery fell within NEST 4. Similarly, neurosurgical procedures included in our study were categorized as NEST 3 [14]. According to the literature, the incidence of MINS ranges from 14% to 17% following hip fracture surgery [39,40], 15% after minor neurosurgical procedures [41], and 12.6% after acute abdominal surgeries [42]. In our cohort, 13% of patients developed MINS, which is consistent with the reported ranges in the literature.

We consider that this created new model is the first in the literature that can be applied to predict MINS in elderly patients who undergo non-elective surgery. Since it is created by using easily obtained parameters, this model should be used in elderly patients undergoing major non-elective surgery to predict MINS. However, we also believe that this model should be validated in large multi-center studies.

## 6. Limitations

The following limitations should be considered when interpreting this study’s findings. First, there is no universally accepted definition of MINS for non-elective patients. Second, troponin was measured within the first 3 days after major surgery and then only in the case of clinical suspicion of acute coronary syndrome or hemodynamic instability. Therefore, a small number of asymptomatic MINS patients may have been missed. Thirdly, although it seems that the type of surgery did not make a statistical difference in the results, combining three different types of surgery might have created a minor limitation. Fourth, this study was conducted at a single center in one geographical location. Thus, our results must be confirmed by large and multi-center studies. Finally, it can be noted as a limitation that other published studies, in addition to the VISION study, were considered for the MINS criteria.

## 7. Conclusions

Based on our study’s findings, a new risk preoperative model is found to successfully predict MINS in elderly patients undergoing non-elective surgery. In addition, this new model is found to be superior to the RCRI in predicting MINS.

## Figures and Tables

**Table 1:** Summary descriptive table by groups of myocardial injury after non-cardiac surgery.

	POST-OP MINS (−)	POST-OP MINS (+)	*p*.Overall
	*N* = 322	*N* = 48	
Age	80.0 [73.2;86.0]	82.5 [76.0;88.0]	0.152
Surgery Type			0.428
1 (Hip Fracture)	217 (67.4%)	28 (58.3%)	
2 (Neurosurgical)	53 (16.5%)	11 (22.9%)	
3 (Acute Abdoman)	52 (16.1%)	9 (18.8%)	
Gender (Male)	132 (41.0%)	19 (39.6%)	0.978
Hypertension	234 (72.7%)	39 (81.2%)	0.278
Diabetes Mellitus	104 (32.3%)	17 (35.4%)	0.791
Cancer History	55 (17.1%)	7 (14.6%)	0.822
COPD	31 (9.63%)	3 (6.25%)	0.597
Stroke History	28 (8.70%)	9 (18.8%)	0.039
Heart Failure	29 (9.01%)	10 (20.8%)	0.025
Coronary Artery Disease	36 (11.2%)	6 (12.5%)	0.980
Insulin Using	56 (17.4%)	7 (14.6%)	0.782
Chronic Kidney Disease	25 (7.76%)	11 (22.9%)	0.003
Revised Cardiac Index	1.00 [0.00;1.00]	1.00 [0.00;2.00]	0.008
White Blood Cell (10^3^/µL)	9.10 [7.21;10.8]	9.35 [6.70;10.8]	0.828
Hemoglobine (g/dL)	11.4 [10.0;12.6]	9.95 [8.90;11.5]	<0.001
Lymphocyte (10^3^/µL)	1.44 [0.94;1.92]	1.11 [0.82;1.55]	0.017
Neutrophil (10^3^/µL)	6.58 [4.97;9.38]	6.96 [4.70;9.93]	0.883
Platelet (10^3^/µL)	224 [178;289]	206 [158;277]	0.221
Creatinine (mg/dL)	1.05 [0.85;1.29]	1.16 [0.88;1.34]	0.283
Urea (mg/dL)	48.0 [35.0;73.0]	74.0 [51.0;112]	<0.001
AST (U/L)	22.0 [17.0;30.0]	26.0 [18.2;36.2]	0.053
ALT (U/L)	16.0 [11.2;22.0]	15.0 [9.00;27.2]	0.741
TSH (mIU/L)	1.35 [0.80;2.11]	1.75 [1.12;2.26]	0.081
Glucose (mg/dL)	124 [104;155]	122 [97.0;146]	0.468
CRP (mg/dL)	46.5 [12.7;102]	49.4 [14.1;101]	0.861
FT4 (ng/dL)	1.03 [0.82;1.26]	0.87 [0.73;1.24]	0.254
Albumin (g/L)	32.0 [28.7;36.0]	30.0 [27.8;34.2]	0.142
D-Dimer (ng/mL)	921 [0.00;2340]	1755 [198;2670]	0.105
EF	60.0 [60.0;61.0]	60.0 [53.8;60.2]	0.006
LVDD (mm)	47.0 [45.0;50.0]	49.0 [45.0;52.2]	0.074
LAAP (mm)	38.0 [36.0;40.0]	40.5 [36.8;44.0]	0.003
MR	49 (15.2%)	13 (27.1%)	0.065
AR	24 (7.45%)	10 (20.8%)	0.006
AS	17 (5.28%)	4 (8.33%)	0.333
MS	13 (4.04%)	3 (6.25%)	0.447
TR	60 (18.6%)	11 (22.9%)	0.612
PASP	6.50 [1.00;12.0]	10.0 [1.00;13.2]	0.167
Follow-up			
ICU Stay (day)	2.00 [0.00;3.00]	6.00 [3.00;11.5]	<0.001
Total Length of Hospital Stay (day)	9.50 [7.00;12.0]	12.0 [8.75;19.0]	0.001
In-hospital Mortality	45 (14.0%)	25 (52.1%)	<0.001

Abbreviations: AS: Aorta Stenosis, AR: Aorta Regurgitation, COPD: Chronic Obstructive Pulmonary Disease, EF: Ejection Fraction, ICU: Intensive Care Unit, LAAP: Left Atrium Anterior Posterior Dimension, LVDD: Left Ventricle Internal Diastolic Dimension, MS: Mitral Stenosis, PASP: Pulmonary Artery Systolic Pressure, TR: Tricuspid Regurgitation.

**Table 2:** Logistic regression for detecting independent predictors of myocardial injury.

Variables	Odds Ratio	95% CI	*p*-Value
Lymphocyte	0.691	0.447–1.068	0.096
Hemoglobin	0.469	0.291–0.758	0.002
Serum creatinine	1.194	1.039–1.372	0.012
D-dimer	2.207	1.231–3.954	0.008
EF	0.938	0.903–0.975	0.001
CVD	2.043	0.819–5.094	0.125
Moderate–severe AR	5.899	2.329–14.940	0.001

Abbreviations: AR: Aorta Regurgitation, CVD: Cerebrovascular Vascular Diseases, EF: Ejection Fraction.

## Data Availability

The datasets generated during and/or analyzed during the current study are available from the corresponding author upon reasonable request. Due to legal and ethical concerns, publication of the data is not preferred.
