# Correction: Cicek et al. A New Risk Prediction Model for the Assessment of Myocardial Injury in Elderly Patients Undergoing Non-Elective Surgery. *J. Cardiovasc. Dev. Dis.* 2025, *12*, 6

**DOI:** 10.3390/jcdd13030125

**Published:** 2026-03-09

**Authors:** Vedat Cicek, Mert Babaoglu, Faysal Saylik, Samet Yavuz, Ahmet Furkan Mazlum, Mahmut Salih Genc, Hatice Altinisik, Mustafa Oguz, Berke Cenktug Korucu, Mert Ilker Hayiroglu, Tufan Cinar, Ulas Bagci

**Affiliations:** 1 Machine & Hybrid Intelligence Lab, Department of Radiology, Northwestern University, Chicago, IL 60611, USA; ulasbagci@gmail.com; 2 Sultan II. Abdulhamid Han Training and Research Hospital, Department of Cardiology, Health Sciences University, 34668 Istanbul, Turkey; babaoglumert11@gmail.com (M.B.); dr.sametyavuz@gmail.com (S.Y.); hatice.altinisik98@gmail.com (H.A.); drmustafaoguz@hotmail.com (M.O.); 3 Van Training and Research Hospital, Department of Cardiology, Health Sciences University, 65300 Van, Turkey; faysalsaylik@gmail.com; 4 Sultan II. Abdülhamid Han Training and Research Hospital, Department of General Surgery, Health Sciences University, 34668 Istanbul, Turkey; a.furkan.mazlum@gmail.com (A.F.M.); mslhgenc@gmail.com (M.S.G.); 5 Department of Internal Medicine, Rutgers\Robert Wood Johnson Barnabas Health, Jersey City Medical Center, Jersey City, NJ 07302, USA; cenkorucu@gmail.com; 6 Department of Cardiology, Dr. Siyami Ersek Cardiovascular and Thoracic Surgery Research and Training Hospital, 34668 Istanbul, Turkey; mertilkerh@gmail.com; 7 School of Medicine, University of Maryland, Baltimore, MD 21201, USA; drtufancinar@gmail.com

In the original publication [1], there was a mistake in “Figure 2” when published. “Figure was replaced by mistake during the proofreading process”. The corrected “Figure 2” appears below. The authors state that the scientific conclusions are unaffected. This correction was approved by the Academic Editor. The original publication has also been updated.


